# The Effectiveness of Metabolic Bariatric Surgery in Preventing Gynecologic Cancer - from Pathophysiology to Clinical Outcomes

**DOI:** 10.7150/jca.91471

**Published:** 2024-01-01

**Authors:** Nikolaos Machairiotis, Athanasios G. Pantelis, Anastasios Potiris, Theodoros Karampitsakos, Petros Drakakis, Eirini Drakaki, Panagoula Oikonomou, Christina Nikolaou, Dimitrios Matthaios, Charalampos Charalampidis, Aris Ioannidis, Paul Zarogoulidis, Stavros Sofoklis

**Affiliations:** 1 Third Department of Obstetrics and Gynecology, National and Kapodistrian University of Athens Medical School, Attikon Hospital,1 Rimini, 124 62 Athens, Greece.; 2 Second Department of Surgery, University Hospital of Alexandroupolis, Medical School, Democritus University of Thrace, Alexandroupolis, Greece.; 3 Oncology Department, General Hospital of Rhodos, Rhodos, Greece.; 4 Pathology Department, University of Cyprus, Cyprus.; 5 Surgery Department, Genesis Private Clinic, Thessaloniki, Greece.; 6 Pulmonary Department, General Clinic Euromedica, Thessaloniki, Greece.

**Keywords:** metabolic bariatric surgery, gynecologic cancer, metabolic rate

## Abstract

Obesity and cancer represent two pandemics of current civilization, the progression of which has followed parallel trajectories. To time, thirteen types of malignancies have been recognized as obesity-related cancers, including breast (in postmenopausal women), endometrial, and ovarian cancer. Pathophysiologic mechanisms that connect the two entities include insulin resistance, adipokine imbalance, increased peripheral aromatization and estrogen levels, tissue hypoxia, and disrupted immunity in the cellular milieu. Beyond the connection of obesity to carcinogenesis at a molecular and cellular level, clinicians should always be cognizant of the fact that obesity might have secondary impacts on the diagnosis and treatment of gynecologic cancer, including limited access to effective screening programs, resistance to chemotherapy and targeted therapies, persisting lymphedema, etc. Metabolic bariatric surgery represents an attractive intervention not only for decreasing the risk of carcinogenesis in high-risk women living with obesity but most importantly as a measure to improve disease-specific and overall survival in patients with diagnosed obesity-related gynecologic malignancies. The present narrative review summarizes current evidence on the underlying pathophysiologic mechanisms, the clinical data, and the potential applications of metabolic bariatric surgery in all types of gynecologic cancer, including breast, endometrial, ovarian, cervical, vulvar, and vaginal.

## Introduction

Obesity is a chronic, relapsing, progressive disease that has been characterized as a pandemic over the last few decades by the WHO 1, 2. This surge in the prevalence of obesity, in turn, has been followed by an exponential increase in metabolic bariatric surgery (MBS), which has been recognized as the most effective intervention for treating obesity and its sequelae with a favorable safety profile 3-5. Among the long-term benefits of MBS, cancer prevention has presumably the most striking impact on shaping the opinion of healthcare providers and patients alike.

Cancer is a multifactorial and multistage disease process, as much as obesity is. The first systematic and comprehensive attempt to correlate obesity (or “body fatness”) with cancer was published in 2016 by the International Agency for Research on Cancer (IARC) and has been updated recently 6, 7. In this context, there has been sufficient evidence that obesity significantly increases the incidence of at least 13 types of cancer, including esophageal adenocarcinoma; cancer of the gastric cardia; colorectal, liver, gallbladder, and pancreatic cancers; breast cancer in postmenopausal women; endometrial and ovarian cancer; renal cell carcinoma, meningioma, and multiple myeloma, with the respective relative risks (RR) ranging from 1.1 to 7.1 (95% CI 1.0-8.1), depending on cancer type (**Table 1**) 7.

This epidemiological correlation has been supported by Mendelian randomization studies, which have showcased the causal effects of visceral adiposity on the manifestation of neoplasia, 8, 9 further confirming the dictum that obesity is not a mere risk factor for developing cancer but a pivotal player of its etiopathogenetic continuum 10. On mechanistic grounds, several processes have been proposed and investigated: the interplay between adipocytes and inflammatory cells via cytokines and reactive oxygen species; the endocrine properties of adipocytes, through both peripheral aromatization of androgens and direct increase of gastrointestinal hormones such as leptin, resistin, and visfatin; the role of the microbiome and its alterations in the context of obesity; the anabolic effects of insulin resistance and diabetes on the survival of cancer cells; genetic predisposition and epigenetic changes to the genome that are accelerated in the context of obesity; etc. 11, 12. In all these processes, the role of adipose tissue and its alterations is central: from inflammation to fibrosis and extracellular matrix remodeling; to an altered microenvironment that affects lipid metabolism and induces insulin resistance; to microbiotal dysbiosis and disrupted immune function; and to imbalanced sex hormone and adipokine secretion 13, 14.

The role of MBS in preventing cancer has recently started to be documented in large-scale population studies with long-term follow-up periods. Among the relevant seminal studies, there are a few worth mentioning. For instance, Schauer et al. retrospectively analyzed the data of 22,198 post-bariatric patients from multiple centers and matched them to 66,427 non-operated individuals over a period of 7 years 15. In the post-bariatric cohort, they found a 33% lower hazard of developing any cancer [hazard ratio (HR) 0.67, 95% CI 0.60-0.74] and an even lower risk of developing obesity-related cancer (HR 0.59, 95% CI 0.51-0.69). Similarly, Aminian et al. retrospectively analyzed a cohort of 30,318 individuals (among whom 5,053 had undergone MBS). They found that those who had undergone MBS featured a significantly lower incidence of obesity-associated cancer (HR 0.68, 95% CI 0.53-0.87), while cancer-related mortality was almost half after MBS (HR 0.52, 95% CI 0.31-0.88) as compared to nonsurgical care 16. In a recent report, Khalid et al. demonstrated that patients who were eligible for (but eventually did not undergo) bariatric surgery had significantly (*p* <0.0005) higher risk for developing any cancer type (4.61%) as compared to patients who were submitted to laparoscopic sleeve gastrectomy (LSG, 3.47%) or Roux-en-Y gastric bypass (RYGB, 3.62%) 17. Several pertinent meta-analyses have also been published (**Table 2**) 18-20. In the most recent one (2023), Wilson et al. analyzed 32 primary studies and found a significant reduction in the overall incidence of cancer (RR 0.62, 95% CI 0.46-0.84), obesity-associated cancer incidence (RR 0.59, 95% CI 0.39-0.90), and cancer-related mortality (RR 0.51, 95% CI 0.42-0.62) 20. Even more compelling, though, is the evidence that stems from the seminal prospective SOS (Swedish Obese Subjects) study. The initial seminal report in 2009 suggested that MBS has a protective role regarding carcinogenesis only for women, not for men 21. The most recent relevant report from the SOS study regarding 701 patients with obesity and diabetes (MBS arm: 393 patients, conventional treatment: 308 patients) showed that, during a median follow-up of 21.3 years (IQR 17.6-24.8, maximum 30.7), the incidence rate of first-time cancer was 9.1/1000 person-years in the bariatric group versus 14.1/1000 person-years in the conventional group (adjusted HR 0.63, 95% CI 0.44-0.89) 22. Interestingly, diabetes remission at 10 years was associated with reduced cancer incidence (adjusted HR 0.40, 95% CI 0.22-0.74), implicating a pivotal role of hyperinsulinemia and insulin resistance in the pathophysiology of obesity-related carcinogenesis 22.

Several studies have focused on the impact of MBS on cancer with regard to female sex. For example, Tsui et al. investigated the risk of developing female-specific cancers following MBS 23. After matching 55,781 post-bariatric females with 247,102 women living with obesity, they found an overall incidence of female-specific cancers of 2.09% in the former (bariatric) group versus 2.69% in the latter (non-bariatric; *p* <0.0001), with a hazard ratio for female-specific cancers of 0.78 (95% CI 0.73-0.83) in the post-bariatric group. Additionally, Adams et al. published their retrospective population study on long-term cancer outcomes following MBS 24. Their study spanned 37 years (1982-2019), investigated 21,837 post-MBS patients matched 1:1 by age, sex, and body mass index (BMI) with non-surgical individuals, and found a 25% overall decrease in the risk of developing cancer (HR 0.75, 95% CI 0.69-0.81). Interestingly, their outcomes were of relevance to the female population, as women demonstrated a reduced overall cancer incidence (HR 0.67, 95% CI 0.62-0.74), obesity-related cancer incidence (HR 0.59, 95% CI 0.52-0.66), and cancer mortality (HR 0.53, 95% CI 0.44-0.64), compared to men. A few years earlier, a meta-analysis of 7 relevant studies demonstrated a total RR of 0.41 (95% CI 0.31-0.56) for developing breast, ovarian, and endometrial cancer after MBS 25. Besides, evidence from the SOS study also supports the notion that women are particularly favored by MBS, since they appear to have a reduced incidence of cancer in general (HR 0.58, 95% CI 0.44-0.77) and gynecologic cancer in particular (HR 0.68, 95% CI 0.52-0.86) 22, 26.

In the present narrative review, we will focus on the potential benefit of MBS in preventing various types of gynecologic neoplasms, including breast, endometrial, and ovarian cancer, as it has been documented in recent publications. Simultaneously, we will present the pathophysiological grounds of the correlation of these cancers with obesity and potential implications in future therapeutic strategies.

## Breast Cancer

Obesity is among the most robustly documented risk factors for the development of breast cancer 27. Interestingly, delving into biological processes will make us realize that obesity serves far beyond a mere risk factor for breast cancer 10. On a phenotypical level, not all breast cancers are the same, nor are they inherently related to obesity. In premenopausal women, there seems to be an inverse association of estrogen receptors (ER^+^ cancers) with obesity (as per BMI and per adiposity), a positive correlation between obesity and triple-negative cancers (43-80% higher risk), and a nonsignificant association between HER2^+^ status and obesity 28. On the contrary, there seems to be an increased risk of ER^+^ tumors, increased incidence of triple-negative breast cancers (TNBC), and worse overall survival in HER2^+^ carriers regarding postmenopausal patients living with obesity who develop breast cancer 28. According to a recent meta-analysis, obesity had a negative impact on disease-free survival (DFS) and overall survival (OS) for all breast cancer subtypes: the hazard ratios (HR) regarding DFS were 1.26 (95% CI 1.13-1.41) for hormone receptor positive/HER2 negative tumors (HR^+^HER2^-^), 1.16 (95% CI 1.06-1.26) for HER2^+^ cancers, and 1.17 (95% CI 1.06-1.29) for TNBC, whereas the respective values regarding OS were 1.39 (95% CI 1.20-1.62) for HR^+^HER2^-^ tumors, 1.18 (1.05-1.33) for HER2^+^ tumors, and 1.32 (95% CI 1.13-1.53) for TNBC 29. Interestingly, these correlations were only applicable to obesity, as no significant association was shown between simply overweight and DFS or OS of breast cancer, possibly suggesting a linear association between the severity of obesity and susceptibility to developing breast cancer.

On a molecular level, the interplay between obesity and breast carcinogenesis is heralded expansion, inflammation, and dysfunction of the adipose tissue 30. These alterations foster at least four major molecular conditions 28, plus newly-discovered ones:

1) Hyperinsulinemia and increased levels of insulin growth factor-1 and -2 (IGF-1 and IGF-1), as a result of high circulating levels of free fatty acids (from increased lipolysis) and glucose (from gluconeogenesis) and the subsequent development of peripheral insulin resistance 31. The same conditions also lead to decreased sex hormone binding globulin (SHBG), which increases the levels of free estrogens 31. In turn, insulin and IGF increase have pleotropic and interactive effects on a subcellular level: i) conjugation of IGF-1 with its receptor (IGF-R) leads to activation of multiple kinase downstream pathways, the end result of which is endocrine-resistant cell growth; ii) there is crosstalk between IGF-R and insulin receptor that has an additive effect on hormonal independence; iii) activation of IGF-R by IGF-2 induces the activation of the epidermal growth factor receptor (EGFR), which attributes proliferation independence to affected cells; and iv) intracellular androgen receptors (AR) and estrogen receptors (ER) induce hormonal independence via IGF-independent activation of the IGF-R 32.

2) Imbalance of adipokines (*aka* adipose-derived cytokines), i.e., increase in leptin, interleukin 6 (IL-6), and tumor necrosis factor alpha (TNF-α), and decrease in adiponectin, which consequently induce the expression of their respective receptors. In turn, conjugation of leptin with its receptor (ObR) leads not only to activation of multiple signal transduction pathways, but also to augmented cross-talk with other receptors, including epidermal growth factor (EGFR), Notch, ER, and interleukin (IL) receptors 32. The end results are multiple: cell proliferation (via cyclin D1), inhibition of apoptosis (via Bcl-2 family and surviving), increase of oncogenic signals (hypoxia-inducible factor-1 or HIF-1a, heat shock protein 90 or Hsp90), modifications of the extracellular matrix and facilitation of metastasis (via metalloproteases or MMPs and serpin), and angiogenesis (via vascular endothelial growth factor or VEGF) 32. On the other hand, low levels of adiponectin lead to differential activation of its receptor via alternative signal transduction (induction of Ras-MAP kinase and mTOR pathways instead of activation of the PGC-1α pathway and inhibition of mTOR) 33.

3) Increased activity of aromatase, which is induced by several pathways (IGF-1, leptin, prostaglandin E2 (PGE2), TNF-α, and LI-1β) and leads to increased estrogen synthesis and promotion of estrogen receptor (ER) expression 32. Furthermore, aromatase activity is amplified by estradiol itself in a positive feedback manner and is further enhanced by inhibition of aromatase dephosphorylation which leads to enduring aromatase action 32. The net effect is an increase in aromatase expression and activity, increase in estradiol production and bioavailability, and enhanced ER activation. Besides, it is evident that there are two pathways for increased estrogen secretion, one via increased lipolysis (see number 1) and one via increased aromatase activity. Increased estrogens, in turn, have three major consequences: i) increased estrogen metabolism which leads to the production of toxic products, such as reactive oxygen species and quinones, ii) increased cellular proliferation that leads to replication stress, and iii) decreased DNA damage repair 34. The cumulative result of these processes is additive DNA damage, which promotes tumorigenesis.

4) Increased synthesis of cholesterol, which leads to defective sterol regulated element binding protein-1 and -2 (SREB-1 and SREB-2) expression and further upregulation of the hydroxylmethylglutatyl-receptor (HMGR) 28. Furthermore, 27-hydorxy cholesterol (27-OHC), a metabolite of cholesterol hydroxylation, has been found to serve as an endogenous selective estrogen receptor modulator (SERM) 35. Additionally, 27-OHC competitive binds to liver X receptor (LXR) and cancels the physiological effect of LXR, which is inhibition of cell proliferation 35.

5) Enhanced function of fatty acid binding protein (FABP4), a protein that facilitates the absorption and utilization of water-insoluble dietary long-chain fatty acids, has been proposed as one of the novel mechanisms that propagates breast cancer development in the context of obesity 36. FABP4 secreted by tumor-associated macrophages and circulating FABP4 secreted by dysfunctional adipocytes leads, via various signal transduction pathways, to enhanced stem cell-like phenotype and tumor progression 36.

6) Micro RNA (miRNA) constitutes another relatively novel mechanism that plays a key role in breast tumorigenesis in the context of obesity. MiRNA is a relatively recently discovered class of RNA regulatory genes with multiple implications on structural, catalytic, and regulatory cellular functions. More than 2500 members of this class of molecules have been discovered and their upregulation or downregulation has been correlated with various disease processes. Relative to our subject, there are breast cancer-associated miRNAs (upregulation of miR-20, downregulation of miR-46), obesity-related miRNAs (upregulation of miR-23, downregulation of miR-14), miRNAs common to breast cancer and obesity (let-7, miR-21, -30c, -31, -93, -124, -143, -155, -181a, -221/222, -326, 335), and miRNAs common in breast cancer and obesity-associated breast cancer (upregulation of miR-302b, downregulation of miR-498) 37. MiRNAs also have different functions: some are related to tumor suppression (i.e., let-7, -200, -205, -145) and are downregulated in the context of breast cancer, some serve as oncogenic signal (miR-10, -17, -21, -155) and are upregulated in breast tumorigenesis, and some propagate metastasis (miR-9, -36, -10b, -37, -38, -21, -39-45, -29a, -46, -373/520) 38, 39.

On a nuclear level, the aforementioned molecular mechanisms converge on three discrete families of transcription factors (TFs): hypoxia-induced factor (HIF), p53, and estrogen receptor 40. Besides, different mechanisms prevail in different cell types. For example, fat-rich diet and hypoxia act directly on immune cells facilitating their activation and the release of inflammatory cytokines (PGE2, IL-6, TNF-α), while at the same time enhance glucose uptake, aerobic glycolysis, and cell proliferation; leptin, IL-6, TNF-α, and PGE2 act on adipose stromal cells and promote glucose uptake, aerobic glycolysis, estrogen production, and cell proliferation via the HIF and p53 families of TFs; finally, insulin, leptin, PGE2, and estradiol act on tumor cells and induce glucose uptake, aerobic glycolysis, protein synthesis, nucleotide synthesis, and cell proliferation primarily via the estrogen receptor family of TFs 40. At the same time, it has recently been shown that increased BMI modifies the levels of tumor-infiltrating lymphocytes (sTILs), thus decreasing pathological complete response (pCR) rates and survival in TNBC patients 41. The key players for orchestrating all these processes and secreting pivotal molecules are adipose tissue-derived mesenchymal stromal/stem cells (ASCs/MSCs) 42, 43. The alterations that these mechanisms bring upon, under the influence of single-nucleotide polymorphisms (SNPs) and epigenetic modifications (obesogenic dietary patterns, unhealthy foods, sedentary lifestyle and lack of exercise), affect all stages of tumorigenesis, including initiation, progression, migration, invasion, and metastasis 28, 30, 38, 39, 44.

**Figure 1:**
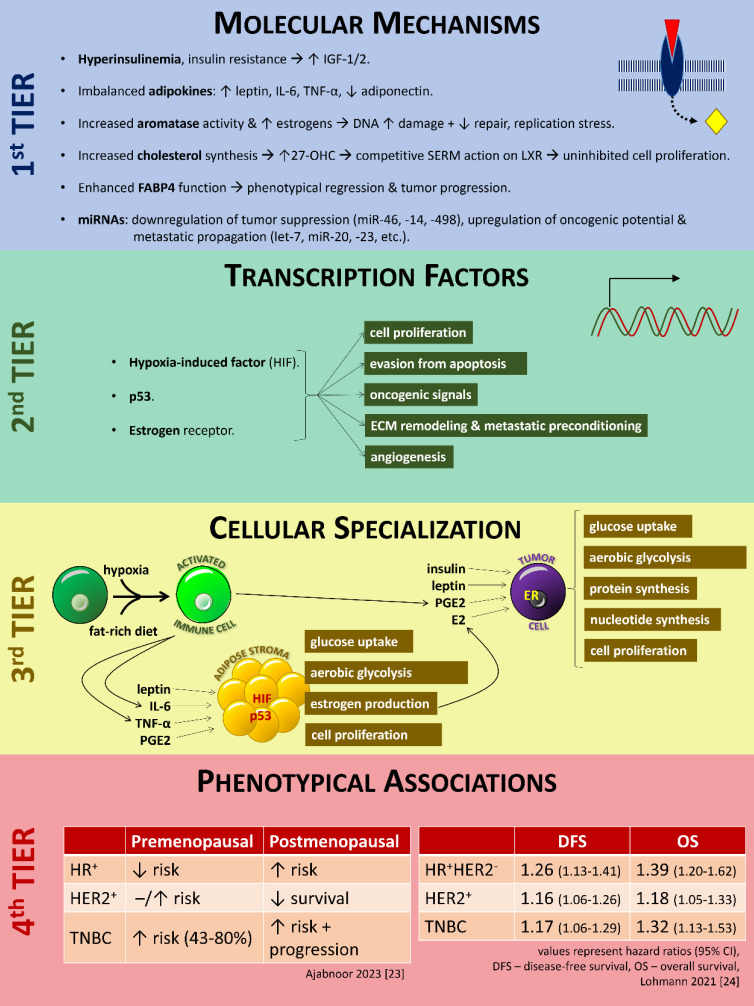
Schematic representation of the current knowledge of the mechanisms linking obesity with carcinogenesis in the breast. For details, please refer to the text. **Key** - ↑, increase; ↓, decrease; -, steady state; IGF-1, insulin-like growth factor-1; IL-6, interleukin-6; TNF-α, tumor necrosis factor-α; 27-OHC, 27-hydroxycholesterol; SERM, selective estrogen receptor modulator; LXR, liver X receptor; FABP4, fatty acid-binding protein-4; miRNA, micro-RNA; PGE2, prostaglandin E2; HIF, hypoxia-induced factor; E2, estradiol; ER, estrogen receptor; HR, hormone receptor; HER2, epidermal growth factor receptor 2; TNBC, triple=negative breast cancer; DFS, disease-free survival; OS, overall survival; CI, confidence interval. Figure designed by A. G. Pantelis, based on evidence described in the main text.

The clarification of these mechanisms (summarized in **Figure 1**) does not have only theoretical implications on the correlation between obesity and breast tumorigenesis. It can also serve as a scaffold to interpret why various interventions to intercept obesity yield clinical benefit on breast cancer prevention, improved response to oncological therapy, and improved prognosis and increased survival after the diagnosis of breast cancer 45.

Beyond its metabolic sequelae (which include type 2 diabetes mellitus and cancer), obesity has further implications regarding mechanical, monetary, and mental issues, as vividly illustrated by A. Sharma (the “4 Ms” of obesity) 46. In this context, there is compelling evidence that obesity constitutes a considerable barrier for patients to participate in appropriate breast cancer screening programs, irrespective of geographical boundaries 47-50. Similar barriers have been also found regarding cervical cancer screening 47-55. Besides, cancer patients who suffer from obesity (and their healthcare providers) must face some additional, more practical challenges. In this context, there are reports that suggest higher recurrence rates in obese patients who undergo breast conserving surgery (BCS) as compared to normal-weight patients. The evidence is even clearer on a less favorable cosmetic outcome in the obesity group after BCS. Obesity has also been linked to more postoperative complications after mastectomy and increased failure rate of sentinel lymph node mapping. Equally challenging is breast reconstruction in obese individuals following mastectomy for breast cancer, owing to high complication rates and suboptimal aesthetic outcome 56. Obesity also has implications in the adjuvant therapeutic setting: patients with large breasts may receive increased doses of radiation, chemotherapy may have increased toxicity and failure rates in the context of obesity irrespective of tumor size, nodal status, and hormone receptor status, whereas aromatase inhibitors may be less effective in overweight and obese populations 56. Eventually, patients living with obesity and breast cancer are at greater risk for developing lymphedema, both before and after mastectomy 56. A pertinent meta-analysis showed that weight-loss interventions lead to a decreased volume of both the affected and unaffected arms but failed to show a significant decrease in the severity of breast cancer-related lymphedema 57.

Dietary modifications, including chronic caloric restriction, time-restricted feeding, fasting, fasting-mimicking diets, intermittent energy restriction, ketogenic diet, and Mediterranean diet, have demonstrated attributes of cancer prevention, restoration of the adipokine balance, improved insulin sensitivity, reduced synthesis of cholesterol and its byproducts, reduced systematic inflammation, and reduced toxicity of chemotherapy 58. Physical activity also seems to have a beneficial impact on modifying the risk of developing breast cancer in women living with obesity 59. Even most importantly, both obesity and interventions to curb obesity seem to affect the prognosis and survival in patients who have already been diagnosed with breast cancer, to opposite directions each 60. In a recent meta-analysis, Pane Y et al. have demonstrated that increased adiposity is linked to significantly elevated all-cause mortality (RR 1.21, 95% CI 1.15-1.27), breast cancer-specific mortality (RR 1.22, 95% CI 1.13-1.32), locoregional recurrence (RR 1.12, 95% CI 1.06-1.18), and distant recurrence (RR 1.19, 95% CI 1.11-1.28) in breast cancer survivors 61. Similarly, worse outcomes regarding DFS and OS have been shown for patients with early breast cancer 62. Interestingly, another meta-analysis demonstrated worse survival rates with increased adiposity based on anthropometric criteria (OS 1.30, 95% CI 1.15-1.46; cancer-specific survival 1.26, 95% CI 1.03-1.55), but failed to prove so as per imaging-measured adiposity 63. Besides, an earlier Cochrane meta-analysis and a recent meta-analysis of randomized controlled trials have shown that weight loss programs (and particularly multimodal interventions including diet, exercise, and psychosocial support) in breast cancer survivors result in significant weight loss and reduction of adipose tissue without increasing adverse effects 64, 65. However, they have also failed to show a clear benefit for survival. This makes the appeal for more radical solutions, such as MBS, very relevant.

Metabolic bariatric surgery (MBS) is the most effective treatment for obesity and related metabolic disorders nowadays 66, and this also holds true regarding the role of MBS in the prevention of breast cancer in this population, according to relevant literature 67. Evidence before 2021 is summarized in a meta-analysis of 11 studies, comprising >1,100,000 patients in total. Breast cancer was diagnosed in 0.54% of post-MBS patients versus 0.84% in controls (RR 0.50, 95% 0.37-0.67). Most importantly, the beneficial effects of MBS were evident for advanced stage disease (stage III or IV, RR 0.50, 95% CI 0.28-0.88), in particular 68. In 2022, Doumouras et al. documented the incidence of breast cancer in 25,448 women (12,724 post-MBS versus an equal number of matched controls): 0.79% in the surgical wing versus 1.09% in the non-surgical one [adjusted HR 0.81 (95% CI 0.69-0.95) at 1 year, 0.76 (95% CI 0.59-0.99) at 7 years] 69. One year later, the same authors retrospectively assessed the risk of breast cancer in a cohort of 69,260 females 70. The non-operated group had a significantly increased hazard for developing breast cancer at 1 year (HR 1.38, 95% CI 1.21-1.58), 2 years (HR 1.31, 95% CI 1.12-1.53), and 5 years (HR 1.38, 95% CI 1.21-1.58). The interesting fact about this study is that the authors estimated the residual risk after MBS, i.e., they compared women who had lost weight with MBS with a sub-cohort of women with BMI <25 Kg/m^2^ 71. In this subgroup analysis, the study failed to show any significant difference in the incidence of breast cancer between the two groups. This observation has multiple implications: further analysis of women who reached a BMI <25 Kg/m^2^ post-MBS is warranted; BMI itself might be a handy index, but is inaccurate and oftentimes misleading as a measure of obesity, as it does not take adiposity into account; breast cancer, as is the case with every cancer, is a multifactorial process, including genetic predisposition, family history, personal history of high-risk lesions or irradiation etc., thus the effect of obesity is obscured by multiple confounders with potentially stronger influence 71-74.

Other authors have investigated the impact of MBS on breast cancer incidence as part of a cumulative investigation regarding gynecologic malignancies. In the meta-analysis of Ishihara et al. the risk of breast cancer was reduced by 49% post-MBS (RR 0.51, 95% CI 0.31-0.83; **Table 2**) 25. Moreover, Tsui et al. demonstrated a breast cancer incidence of 1.50% in the surgical group (N = 55,781) versus 1.75% in the non-surgical group (N = 247,107, *p* <0.0001) 23.

Several studies have proceeded to further analysis of the impact of MBS on breast cancer according to receptor status. When comparing 2,430 post-MBS patients to 2,430 matched non-surgical females, Hassinger et al. found reduced overall breast cancer incidence (0.7% versus 1.3%, *p* = 0.03), lower incidence of invasive breast cancer (0.6% versus 1%), and lower incidence of ER^+^ tumors (36.4% versus 70%, *p* = 0.04) in the post-bariatric group 75. Post-MBS patients also featured lower rates of PR^+^ and higher rates of HER2^+^ cancers, but these differences were non-significant. Furthermore, in a retrospective analysis of 301 pre-menopausal and 399 post-menopausal post-bariatric women compared to 53,889 non-bariatric controls, Feigelson et al. found a 37% reduction in the overall risk of breast cancer after MBS. Moreover, they showed that ER^+^ tumors were less prevalent in the post-bariatric group, but this finding was significant only for postmenopausal women (premenopausal: HR 0.84, 95% CI 0.62-1.13; postmenopausal: HR 0.52, 95% CI 0.39-0.70) 76. Besides, Heshmati et al. showed that post-MBS patients have lower risk of developing HER+ tumors as compared to their non-operated counterparts (OR 0.16, 95% CI 0.03-0.76). Interestingly, this study did not show any difference between groups regarding hormone receptor status 77. Likewise, the recent study of Doumouras et al. failed to show any significant difference between the bariatric group and the various BMI subgroups regarding hormone receptor and HER2 status 70.

The role of MBS as secondary prevention after the manifestation of breast cancer deserves special mention, as vigorous pertinent research is underway. In a case series of 13 patients, Zhang et al. found that MBS following the diagnosis and treatment of breast cancer at a median interval of 3 years is feasible and safe 78. In a much larger, population-based study of 395,146 breast cancer survivors, Lee et al. found that MBS that was performed after the diagnosis of cancer was associated with a non-significant decrease in mortality (cause-specific HR 0.48, 95% CI 0.15-1.53). However, after adjustment for age, stage, comorbidity, race/ethnicity and socioeconomic status, post-diagnosis MBS was associated with a decreased mortality risk (HR 0.37, 95% CI 0.01-0.99) in the entire cohort, that also comprised endometrial cancer survivors 79. Despite the inherent methodological limitations of this study (i.e., survivorship bias owing to its retrospective design), it can serve as a primer for future investigation on the benefits of MBS as a measure to radically improve survival following breast and other obesity-related cancers 80. Besides, there is initial evidence that MBS might improve the response to therapy for breast cancer. Sipe et al. investigated the role of sleeve gastrectomy with regards to the response to immune checkpoint blockage in a rodent animal model and found that surgical weight loss followed by immunotherapy with anti-PDL-1 (anti-programmed death ligand-1 antibodies) in formerly obese mice resulted in reduced cancer burden and favorable locoregional immune milieu 81. As noted earlier, relevant research on the role of MBS in improving the disease burden in breast cancer survivors is still ongoing and a promising future on expanding the indications of MBS lies ahead 67.

In summary, there is adequate evidence that obesity is etiologically linked to breast cancer rather than merely serving the role of a risk factor. Metabolic bariatric surgery consistently seems to be an effective intervention for curbing the risk of developing breast cancer in patients living with obesity, whereas emerging evidence suggests that MBS could radically improve the prognosis in breast cancer survivors.

## Endometrial Cancer

Cancer of the uterine corpus along with (postmenopausal) breast cancer, share the first two positions of the most common obesity-related malignancies, by incidence in the general population 82. According to IARC, endometrial cancer bears the highest relative risk among obesity-related cancers (7.1, 95% CI 6.3-8.1). For the purposes of this review, we will focus on the relationship of endometrial cancer with obesity and its management.

Obesity exerts its effects on the endometrium via three processes primarily, i.e., increased insulin (secondary to insulin resistance), increased aromatase, and imbalanced adipokines (increased leptin, decreased adiponectin), pretty much as is the case with the breast 83, 84. Insulin increases the levels of bioactive IGF-1, both directly in the circulation and indirectly in the endometrium, via a decrease in the IGF-binding globulin (IGFBP) 83. Circulating IGF-1 stimulates the production of androgens by the ovary. This creates a condition of chronic anovulation with a subsequent decrease in progesterone and adiponectin, which also negatively affect IGFBP production in the endometrium 83, 85. Eventually, insulin decreases SHBG, which, along with aromatase, increases the levels of bioactive estrogens. These estrogens increase the levels of IGF1 in the endometrium. Taken together, high IGF1 and low IGFBP in the endometrium lead to increased levels of bioactive IGF1 and this has a hyperplastic and subsequently dysplastic and carcinogenic effect on the endometrium, via the RAS-MAPK and PI3K-AKT-mTOR signal transduction pathways 83, 84, 86. Normally, adiponectin has an inhibitory effect on the AKT/mTOR signal transduction, but in the context of obesity its low circulating levels lead to attenuation of this phenomenon 87. Additionally, leptin binds to its ligand on the endometrial cell and exerts its biologic effects via the JAK/STAT pathway 88.

**Figure 2:**
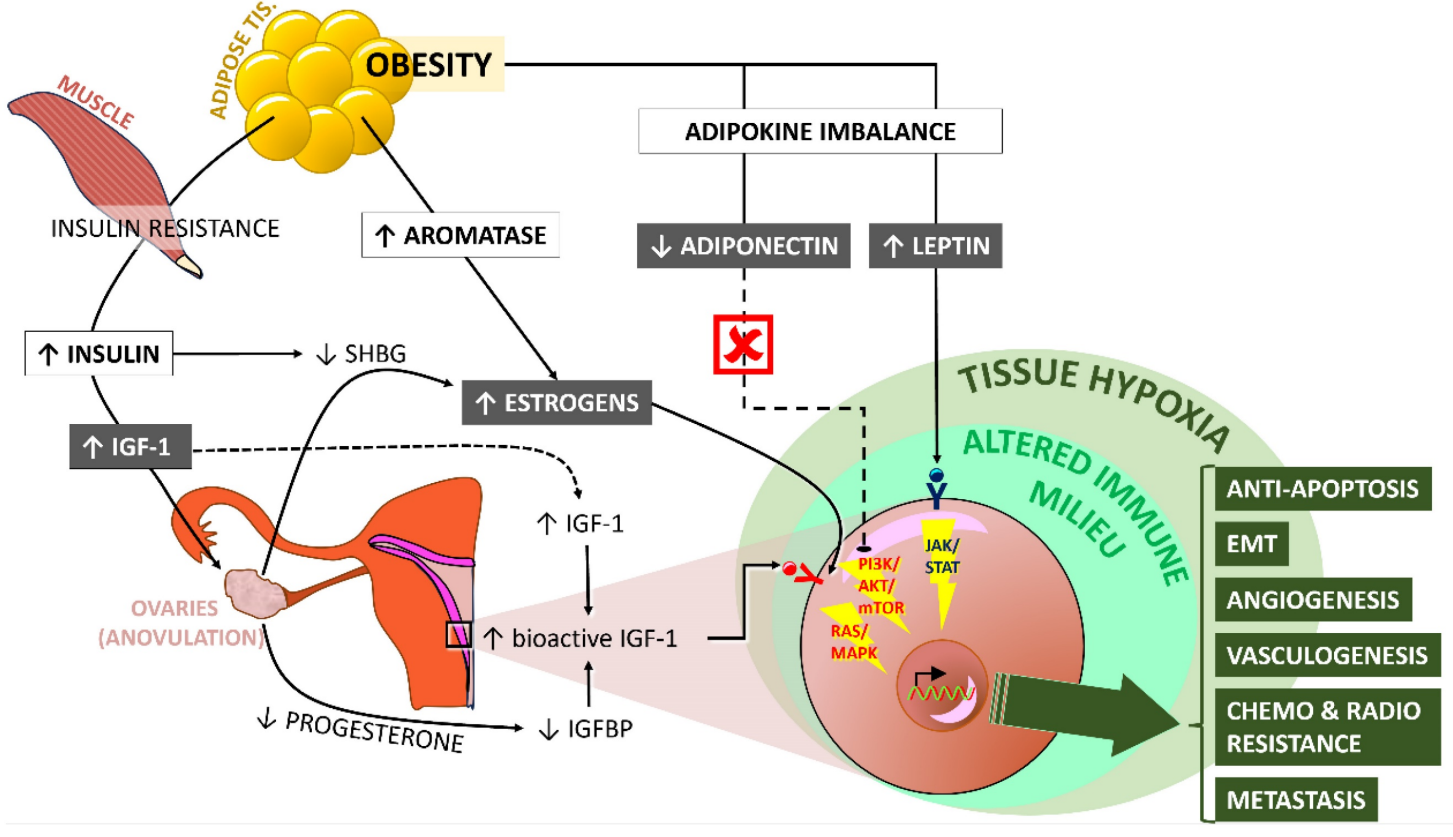
Schematic representation of the mechanisms that connect obesity with carcinogenesis in the endometrium. For details, please refer to the text. **Key** - ↑, increase; ↓, decrease; IGF-1, insulin growth factor-1; SHBG, sex hormone-binding globulin. The ý symbol indicates abolishment of the specific pathway. Figure designed by A. G. Pantelis, based on evidence described in the main text.

All these processes take place and prosper in a local and systematic environment of altered immunity. Studies have demonstrated a link between obesity, endometrial cancer, and increased levels of CRP, IL-1Rα, IL-6, and tissue CD8+ cells. Most importantly, the levels of these components of immunity seem to return back to normal upon effective weight loss following bariatric surgery 89, 90. A condition of paramount importance for the development of this microenvironment seems to be hypoxia. Tissue hypoxia upregulates HIF-mediated transcription and has pleiotropic sequelae related to adverse prognosis in the context of endometrial cancer (as well as other cancer types), including increased cell proliferation; stemness, epithelial-mesenchymal transition (EMT) and aggressive phenotype; metabolic adaptation and drug efflux resulting in resistance to chemotherapy; vasculogenesis (vasculogenic mimicry) and angiogenesis (vascular remodeling); and finally invasive and metastatic potential 91-93. The role of miRNA has also started to be investigated in the context of obesity-related endometrial cancer and relevant research is still ongoing 94. In brief, the etiopathogenetic similarities to obesity-related breast cancer are obvious. **Figure 2** graphically recapitulates the available evidence on the underlying mechanisms that connect obesity and endometrial tumorigenesis.

Clinical evidence supports basic science in the association of obesity with endometrial cancer. Shaw et al. found a pooled effect estimate (pEE) of 2.32 (95% CI 2.09-2.58) among case-control studies, 2.49 (95% CI 2.27-2.73) among cohort studies, and 2.65 (95% CI 2.42-2.90) in total for patients living with obesity 95. The respective figures were 6.54 (95% CI 4.98-8.35), 3.74 (2.94-4.76), and 4.84 (95% CI 3.92-5.97) in patients suffering from severe obesity, indicating a linear relationship between the risk for developing endometrial cancer with increasing body weight. Similar numbers were observed when the risk of endometrial carcinogenesis was analyzed by adiposity [pEE = 2.30 (95% CI 1.71-3.09), 1.92 (95% CI 1.57-2.35), and 1.43 (1.33-1.54), although the magnitude of the effect was a bit lower compared to body weight metrics. Importantly, BMI was associated with increased all-cause and endometrial cancer-specific mortality, particularly in the group of those suffering from severe obesity (pEE = 2.06 (95% CI 1.55-2.74) 95. Another recent meta-analysis of 11 cohort studies by the Epidemiology of Endometrial Cancer Consortium, with 14,859 case and 40,895 controls, found a positive correlation of obesity in adulthood (OR 2.85, 95% CI 2.47-3.29) and early adulthood (OR 1.26, 95% CI 1.06-1.50) with the risk of endometrial cancer 96. These outcomes seem to be generally universal across different ethnic groups, regarding both clinical metrics (BMI, waist circumference) and endometrial cancer-related biomarkers (IGF-1, leptin, adiponectin, IL-1, IL-6) 89, 97-100. Additionally, Wise et al., in a meta-analysis of 3 case-control studies, found that a BMI ≥30 Kg/m^2^ is significantly associated with endometrial cancer in premenopausal women, warranting increased awareness in this age group regarding the beneficial role of losing weight for potentially preventing the manifestation of endometrial cancer 101. In this regard, it is well known that premenopausal and postmenopausal endometrial cancers are two biologically distinct entities. The former manifest at a younger age and are linked to obesity, lipid, and metabolic disorders, are estrogen-dependent and related to a thickened endometrium, bear endometrioid histology, have molecular associations with PTEN, MSI, PI3K/AKT, and KRAS, and generally portend a good prognosis. Conversely, the latter manifest at an older age, are remotely associated with obesity, are estrogen-independent and associated with atrophic endometrium, have poor differentiation, are linked to p53, Her2, PI3/AKT, and KRAS, and have an overall worse prognosis 86, 102. Nevertheless, data from the Women's Health Initiative, comprising 86,937 postmenopausal individuals, showed that an increased risk of endometrial cancer was evident in women with elevated BMI (HR 1.76, 95% CI 1.41-2.19) and waist-to-hip ratio (WHR; HR 1.33, 95% CI 1.04-1.70), thus defying the notion that postmenopausal endometrial cancer is not related to obesity 103.

An interesting element that results from these studies is the defective role of BMI as a metric of obesity. Population-based studies have demonstrated a discrepancy between linear and non-linear models of predicting the incidence of endometrial cancer among women living with obesity. This non-linearity can be attributed to growth-promoting threshold effect (i.e., “second hit” mechanisms beyond the established ones boost the incidence of carcinogenesis after a certain BMI value), loss of regulatory inhibitory effect (this “second hit” mechanism(s) abolishes the inhibitory mechanisms that keep the initiation mechanisms under control), multiplicative interaction (i.e., the underlying mechanisms act synergistically and the final outcome is greater than the mere addition of the individual parts), or “treatment effect” secondary to vigilance and aggressive prevention towards severe obesity but not towards overweight and low-stage obesity 104. Despite these concerns, a recent study showed that uterine cancer was among those malignancies for which BMI was an accurate predictor based electronic health records and prespecified cut-off points 105.

Obesity is not only a risk factor for developing endometrial cancer but might have an impact on prognosis and survival after the diagnosis of endometrial cancer. Although there is evidence that obesity increases cardiovascular and all-cause mortality in endometrial cancer survivors 106, 107, there are conflicting reports regarding its impact on disease progression. For instance, some authors have claimed that obesity is linked to improved DFS in advanced-stage (3 and 4) non-endometrioid endometrial cancer 108, whereas other publications have stated the exact opposite 109. Two recent studies attempt to shed light with regards to the impact of obesity on survival after the diagnosis of endometrial cancer. Lees et al. showed that, among other examined risk factors, obesity itself leads to an increased all-cause mortality (HR 1.77, 95% CI 1.36-2.31), but is not related to cardiovascular or endometrial cancer-specific mortality (95% CI 0.92-2.32 and 0.83-3.93, respectively) 110. Besides, Kokts-Porietis observed that an increase of BMI of >5% within 1 year before the diagnosis of endometrial cancer results in a twofold decrease in OS and DFS in endometrial cancer patients 111. In brief, the impact of obesity on cancer-specific survival is a field of ongoing investigation, as current evidence does not suffice for drawing safe conclusions. However, given the negative impact of obesity on overall survival warrants increased vigilance. In this context, there is great interest in the role of increasing awareness about the risks of obesity in high-risk women and survivors of endometrial cancer, and most importantly in the value of attenuating obesity as a means of secondary prevention against cancer recurrence. A study of 93 women (mean age 44.9 years, mean BMI 48.7 Kg/m^2^) who had enrolled to a bariatric surgery program found that, although 66% of the participants acknowledged that obesity is a risk factor for uterine carcinogenesis, less than half (48%) identified themselves as being at risk, although they suffered from obesity themselves 112. Conversely, another study found that endometrial cancer survivors failed to correctly classify their weight, as only 32% of the participants in the BMI range of 30-34.0 Kg/m^2^ and 72.7% in the BMI range of 35-39.9 Kg/m^2^ identified themselves as living with obesity 113. Haggerty et al. yielded similar results in their survey, in which one third of participants declared being unaware of any association between obesity and endometrial cancer 114. However, 59% were eager to follow a weight loss intervention, pointing out a potential opportunity for weight management in this population group 114. A comparable rate of interest in MBS (61.2%) was documented more recently by Wiley et al. 115. An effective strategy that has been suggested to increase endometrial cancer patient awareness regarding obesity is quality improvement through structured multidisciplinary programs 116. Besides, Njoku et al. have acknowledged that there are several gaps in all tiers of the linkage between obesity and endometrial cancer, from estimating the actual risk to implementing risk-reducing interventions, and from understanding the underlying pathophysiologic mechanisms to implementing established prevention measures, including MBS 117.

MBS seems to be an effective measure against the manifestation and progression of endometrial cancer. On molecular grounds, it has been shown that MBS restores the levels and function of key players of endometrial tumorigenesis, including (in accordance with the mechanisms described earlier) biomarkers of cellular proliferation (Ki-67), signal transduction (pAKT), insulin resistance (HbA1c, HOMA-IR), and inflammation (CRP, IL-6) 118. An additional benefit shown in this study was the restoration of fertility, as it was documented by the normalization of luteinizing hormone (LH), follicle stimulating hormone (FSH), and SHBG 118. On clinical grounds, several relevant studies and meta-analyses have been published 119-121. One of the first papers that denoted the benefits of MBS on reducing the risk for endometrial pathology and is worth mentioning because of it prospective design is the study by Argenta et al. in 59 women who underwent MBS and in whom endometrial biopsy was obtained 122 In this study, the prevalence of occult endometrial pathology was 6.8% at the time of MBS and 6.5% at 1-year follow-up, with resolution of hyperplasia in 2 women, persistent hyperplasia in another 2, and *de novo* hyperplasia in 1 122. Later, the seminal SOS study showed that endometrial cancer was the only gynecologic cancer that had a statistically significant long-term benefit following MBS (HR 0.56, 95% CI 0.35-0.89), although all female cancers (except cervical) were linked to reduced incidence after bariatric surgery (but without statistical significance) 26. In the most recent meta-analysis of 7 relevant index studies by Ishihara et al., the risk of endometrial cancer was reduced by 67% after MBS (RR 0.33, 95% CI 0.21-0.51; **Table 2**) 25. Additionally, in the study of Tsui et al. the incidence of endometrial cancer was 0.47% in the surgical group (N = 55,781) versus 0.76% in the non-surgical group (N = 247,107, *p* <0.0001) 23. More recently, Khalid et al. showed that the incidence of endometrial cancer was significantly higher in non-operated females living with obesity as compared to those who had undergone LSG or RYGB (0.86% versus 0.56% versus o.60%, *p* = 0.007) after 5 years of follow-up. The respective OR was 0.65 (95% CI 0.46-0.92) for LSG and 0.70 (95% CI 0.50-0.98) for RYGB 17. Notably, there is a recent report that defies the benefit of MBS in the incidence of endometrial cancer and disease-specific survival, however it should be acknowledged that the retrospective nature of the study in combination with the small sample size do not allow for generalization of the conclusions 123. Equally dubious are the results of a relevant systematic review regarding the impact of MBS on endometrial hyperplasia; the authors recognize the scarcity of data along with its poor quality 124.

Despite the conflicting data on the role of obesity in disease progression of endometrial cancer survivors, the role of MBS as a secondary prevention measure has started to be investigated. In a recently published case series of 5 patients with endometrial cancer diagnosis, all patients experienced regression of their cancer within 6 months following MBS, along with other obesity-related medical problems 125. The much larger population-based study by Lee et al. that was mentioned earlier for breast cancer, also investigated the role of MBS as a secondary prevention intervention for 69,859 survivors of endometrial cancer. The reduction in mortality risk for endometrial cancer was also non-significant (HR 0.23, 95% CI 0.03-1.70), but it should be reminded that the mortality risk of the cohort overall was decreased 79. In brief, further investigation is warranted to validate the impact of MBS on survivorship following the diagnosis of endometrial cancer.

In brief, evidence shows a mechanistic correlation between obesity and the manifestation of endometrial cancer. Metabolic bariatric surgery is an effective measure for preventing the development of endometrial cancer, especially in high-risk women, but its role as an intervention to extend survival after the diagnosis of endometrial cancer remains elusive.

## Ovarian Cancer

Ovarian cancer is the third gynecologic cancer that is recognized to have clear association with obesity, according to IARC 7. The underlying mechanisms again fit the pattern described earlier for endometrial and breast cancer including hyperglycemia, insulin resistance and IGF-1, deranged adipokine levels (increased leptin, TNF-α, interleukins, decreased adiponectin), inflammatory cytokines and VEGF, and altered levels of steroid hormones 126, 127. Nevertheless, determinants of which phenotype (i.e., endometrial, breast or ovarian cancer) will manifest in each patient remain to be discovered, although genetic predisposition, SNPs, epigenetic factors and the metabolomic milieu obviously play an important role in this regard. For example, there is some evidence that dysregulated lipid synthesis and metabolism has a role in increasing ovarian tumorigenesis in the context of obesity 127.

On clinical grounds, evidence is conflicting, as it is noted by a systematic review of 43 studies with almost 3.5 million participants: 14 studies found a significant correlation between obesity and ovarian cancer, 26 studies failed to show any such association, whereas 3 studies found an inverse relationship between the two entities 128. A recent comprehensive meta-review of systematic reviews and meta-analyses attempted to investigate all factors that are potentially associated with the development of ovarian carcinogenesis. Obesity and overweight were identified in 5 studies collectively among 226 included reviews in total, the former bearing a RR of 1.27 (95% CI 1.19-1.36, I^2^ 0%) and the latter 1.07 (95% CI 1.04-1.10, I^2^ 0%) 129. The role of obesity in ovarian cancer survival has also been investigated by two meta-analyses, which found comparable relative risks with regards to survival between individuals living with obesity and ones with normal-range BMI, but with different statistical significance, according to the included studies (HR 1.17, 95% CI 1.03-1.34 versus 1.11, 95% CI 0.97-1.27) 130, 131. Moreover, there was a inversely proportional relationship between survival and incremental increase of BMI 131. On the contrary, in the meta-analysis by Cheng et al., no correlation was found between imaging-measured adiposity and overall or progression-free survival of ovarian cancer 63.

Data on the effect of MBS on the development of ovarian cancer stems from collective gynecologic cancer studies. According to the seminal SOS study, MBS had the strongest inverse effect on the incidence of ovarian cancer among all gynecologic cancers in the long run, but this effect was not statistically significant (HR 0.51, 955 CI 0.24-1.10) 26. The meta-analysis of Ishihara et al. demonstrated a similar reduction of 53% in the risk for ovarian cancer, but their outcome reached statistical significance (RR 0.47, 95% CI 0.27-0.81, I^2^ 0%) 25. Furthermore, the population-based study of Khalid et al. found an ovarian cancer incidence of 0.43% in non-operated females versus 0.18% post-LSG and 0.15% post-RYGB (*p* = 0.001), resulting in a risk reduction of 58% (OR 0.42, 95% CI 0.24-0.73) for LSG and 66% (OR 0.34, 95% CI 0.19-0.63) for RYGB 17. Finally, Tsui et al. found an ovarian cancer incidence of 0.18% in the post-bariatric group versus 0.28% in non-operated women (*p* <0.0001), with the individual incidences being 0.06% after LSG and 0.09% after RYGB (*p* = 0.0283) 23.

This data shows that ovarian cancer is underrepresented in current literature as compared to breast and endometrial cancer, with regards to its correlation with obesity and the impact of MBS on its incidence and survival. Available evidence has demonstrated potential benefit from MBS, an observation that needs to be validated by dedicated ovarian cancer-oriented studies.

## Closing remarks

This extensive review of pathophysiological mechanisms and recent evidence on the impact of obesity and metabolic bariatric surgery on the development, the progression, and the prognosis of gynecologic malignancies has drawn the following conclusions:

- At present, breast, endometrial, and ovarian cancers are considered established obesity-related neoplasms.
- The relationship between obesity and obesity-related gynecologic cancers is beyond that of a mere risk factor - evidence from basic research and epidemiological projections support the claim that obesity and tumorigenesis are inherently connected at a molecular and pathophysiologic level.
- Insulin resistance and increased IGF-1, disruption of the adipokine equilibrium with increased leptin, interleukins, and TNF-α and decreased adiponectin, augmented peripheral aromatization and production of estrogens, tissue hypoxia and locally disrupted inflammation, and a pivotal role of mesenchymal adipose tissue cells seem to be core motifs in the pathogenesis of obesity-related gynecologic cancers.
- Beyond the connection of obesity with certain types of gynecologic cancer (i.e., the *metabolic* complications of obesity), the clinician should also keep a mind to other potential sequelae of obesity (i.e., *mechanical*, *mental*, and *monetary* complications). In this regard, obesity might represent a substantial obstacle in the access of women to effective screening programs, or might have secondary effects such as resistance to chemotherapy and targeted therapies, persisting lymphedema, etc.
- Metabolic bariatric surgery can serve as a primary prevention measure against obesity-related gynecologic cancers in high-risk female populations and generally women living with obesity.
- Most importantly, metabolic bariatric surgery might hold a pivotal role as a secondary prevention measure in increasing disease-specific and overall survival in patients already diagnosed with obesity-related gynecologic cancers. Reinforcement of relevant evidence will potentially lead to expansion of the indications of MBS and add a safe, effective, and robust intervention in the armamentarium of healthcare professionals who deal with oncologic patients in the context of multidisciplinary management.
- Interpretation of the above-mentioned correlations should be careful, given the retrospective nature of the majority of relevant studies, particularly when it comes to the role of MBS as a measure of secondary prevention in patients who have already manifested one of the gynecologic cancers with an established link to obesity. Ideally, carefully designed randomized trials could establish a causal relationship between MBS and an increase of survivorship after gynecologic cancer. However, we acknowledge the technical difficulty, the considerable cost, and the potential ethical issues of carrying out such meta-analyses in cancer survivors. The implementation of novel research methods, such as the analysis of big data contained in population-based studies and registries with machine learning 132, 133, might serve as reliable alternatives for investigating the role of MBS in preventing gynecologic cancer recurrence and extending survivorship.

## Figures and Tables

**Table 1:** Gynecologic cancers that are linked to obesity, according to the International Agency for Research on Cancer (IARC) 7.

Obesity-related type of gynecologic cancer	RR	95% CI
Breast (postmenopausal)	1.1	1.1-1.2
Corpus uteri (endometrial)	7.1	6.3-8.1
Ovary	1.1	1.1-1.2

**Table 2:** Published systematic reviews and meta-analyses on the relationship between metabolic bariatric surgery and the subsequent manifestation of cancer. All the analyses included population-based (prospective and retrospective) primary studies. All meta-analyses showed a significant decline in overall cancer incidence and obesity-related cancer incidence and/or cancer mortality after bariatric surgery, as compared to matched controls. However, the heterogeneity in most analyses was high. Gynecologic and female-related cancer incidences are highlighted in light grey. Significant associations are written in bold font. **Key** - no., number; incl., included; RoB, risk of bias; publ., publication; OR, (pooled) odds ratio; RR, relative risk; CI, confidence interval; OCI, overall cancer incidence; ORCI, obesity-related cancer incidence, CRC, colorectal cancer; PDAC, pancreatic adenocarcinoma; premen., pre-menopausal; postmen., post-menopausal; HCC, hepatocellular carcinoma; N/A, not available.

First author	Year of publication	Years of publication of included studies	No. of incl. studies (N)	Post-bariatric group (N)	Matched controls (N)	Assessment for…		Outcome	OR/RR	95% CI	Hetero-geneity (I^2^ %)
						RoB	Publ. bias				
Wiggins T	2018	2010 - 2018	8	114,020	521,622	YES	YES	**OCI**	**0.72**	**0.59-0.87**	73.5
								**ORCI**	**0.55**	**0.31-0.96**	97.6
								**Breast**	**0.50**	**0.25-0.99**	94.9
								Endometrial	0.47	0.08-2.65	97.9
								CRC	1.39	0.96-2.02	72.7
								Esophageal	0.79	0.43-1.44	0.0
Zhang K	2019	2004 - 2019	21	304,516	8,492,408	NO	YES	**OCI**	**0.56**	**0.48-0.68**	93.1
								**Ca mortality**	**0.56**	**0.41-0.75**	87.3
								**Breast**	**0.49**	**0.33-0.72**	N/A
								**Endometrial**	**0.43**	**0.26-0.71**	N/A
								CRC	0.82	0.41-1.64	N/A
								PDAC	0.70	0.24-2.01	N/A
Ishihara BP	2020	until 2019	7	150,537	1,461,938	YES	YES	**Overall gynecologic cancer incidence**	**0.41**	**0.31-0.56**	90.0
								**Breast**	**0.51**	**0.31-0.83**	92.0
								**Ovarian**	**0.47**	**0.27-0.81**	0.0
								**Endometrial**	**0.33**	**0.21-0.51**	88.0
Wilson RB	2023	2007 - 2023	32	511,585	1,889,746	YES	YES	**OCI**	**0.62**	**0.46-0.84**	99.0
								**ORCI**	**0.59**	**0.30-0.90**	99.0
								**Ca mortality**	**0.51**	**0.42-0.62**	0.0
								**Breast (overall)**	**0.56**	**0.44-0.71**	96.0
								Breast (premen.)	0.88	0.74-1.04	43.0
								Breast (postmen.)	0.46	0.18-1.18	98.0
								**Endometrial**	**0.38**	**0.26-0.55**	94.0
								**Ovarian**	**0.45**	**0.31-0.64**	74.0
								**CRC (overall)**	**0.69**	**0.53-0.88**	91.0
								**CRC (male)**	**0.65**	**0.43-0.96**	100.0
								CRC (female)	0.75	0.49-1.13	100.0
								**HCC**	**0.35**	**0.22-0.55**	88.0
								**PDAC**	**0.52**	**0.29-0.93**	91.0
								**Gallbladder**	**0.41**	**0.18-0.96**	73.0
								**Kidney**	**0.69**	**0.47-0.99**	96.0
								Esophageal	0.66	0.34-1.30	85.0
								Gastric	0.60	0.21-1.71	89.0
